# Appetite loss and associated factors at 1 year after intensive care unit elder survivors in a secondary analysis of the SMAP-HoPe study

**DOI:** 10.1038/s41598-023-28063-8

**Published:** 2023-01-19

**Authors:** Mio Kitayama, Takeshi Unoki, Aki Sasaki, Hideaki Sakuramoto, Sakura Uemura, Takahiro Tsujimoto, Takako Yamaguchi, Yuko Shiba, Mayumi Hino, Tomoki Kuribara, Yuko Fukuda, Takumi Nagao, Masako Shirasaka, Junpei Haruna, Yosuke Satoi, Yoshiki Masuda

**Affiliations:** 1 Nursing Department Heart Center, Kanazawa Medical University Hospital, Uchinada, Ishikawa, Japan; 2 Department of Acute and Critical Care Nursing, School of Nursing, Sapporo City University, Chuo-ku, Sapporo, Hokkaido Japan; 3 Department of Critical Care and Disaster Nursing, Japanese Red Cross Kyushu International College of Nursing, Munakata, Fukuoka Japan; 4 Emergency and Critical Care Medical Center, Osaka City General Hospital, Osaka, Japan; 5 Resource Nurse Center, Nara Medical University Hospital, Kashihara City, Nara Japan; 6 Intensive Care Unit, Nippon Medical School Musashikosugi Hospital, Kawasaki, Kanagawa Japan; 7 Intensive Care Unit, University of Tsukuba Hospital, Tsukuba, Ibaraki Japan; 8 Intensive Care Unit, Tohoku Medical and Pharmaceutical University Hospital, Sendai, Miyagi Japan; 9 Intensive Care Unit, Jichi Medical University Hospital, Yakushiji, Shimotsuke-shi, Tochigi Japan; 10 Intensive Care Unit, Sakakibara Heart Institute, Fuchu-Shi, Tokyo Japan; 11 Intensive Care Unit & Cardiac Care Unit, Japanese Red Cross Fukuoka Hospital, Fukuoka, Japan; 12 Intensive Care Unit, Sapporo Medical University Hospital, Sapporo, Hokkaido Japan; 13 Intensive Care Unit, Naha City Hospital, Naha, Okinawa Japan; 14 Department of Intensive Care Medicine, School of Medicine, Sapporo Medical University, Sapporo, Hokkaido Japan

**Keywords:** Health care, Medical research

## Abstract

Appetite loss, a common but serious issue in older patients, is an independent risk factor for sarcopenia, which is associated with high mortality. However, few studies have explored the phenomenon of appetite loss after discharge from the intensive care unit (ICU). Therefore, we aimed to describe the prevalence of appetite loss and relationship between appetite loss and depression in patients living at home 12 months after intensive care. This study involved secondary analysis of data obtained from a published ambidirectional study examining post-intensive care syndrome 12 months after discharge (SMAP-HoPe study) conducted in 12 ICUs in Japan. We included patients aged > 65 years. The Short Nutritional Assessment Questionnaire and Hospital Anxiety Depression Scale were used for the analysis. Descriptive statistics and a multilevel generalized linear model were used to clarify the relationship between appetite loss and depression. Data from 468 patients were analyzed. The prevalence of appetite loss was 25.4% (95% confidence interval [CI], 21.5–29.4). High severity of depression was associated with a high probability of appetite loss (odds ratio, 1.2; 95%CI, 1.14–1.28; p = 0.00). Poor appetite is common 12 months after intensive care and is associated with the severity of depression.

## Introduction

Appetite loss in the elderly is a serious issue. The loss of appetite experienced by elderly has been largely attributed to the aging process and it causes sarcopenia^1,2^. A recent systematic review indicated that sarcopenia in the elderly is associated with increased mortality^3^.Thus, Appetite loss in the elderly can have serious consequence.

Most studies of appetite loss related to intensive care have focused on short-term appetite during the intensive care unit (ICU) stay and after discharge, and ICU patients are known to have appetite loss, at least for a short period of time during and after ICU stay^4,5^. However, there is limited research on appetite loss over a longer period, e.g., more than 6 months after ICU discharge^4^.

A previous observational study in a population studied 3 months after ICU discharge indicated that 78.9% of patients reported a decrease in appetite compared to that before ICU admission^5^. Furthermore, another observational study, conducted 6 months after ICU discharge, matched patients with the general population by age and sex, reported that patients after ICU discharge had a lower appetite than the general population. However, these past studies have some issues. First, both studies did not use a validated appetite screening tool. Secondly, the later study had variation in time of 3 months to 3 years at the period between ICU discharge and the point at which appetite was measured. To the best of our knowledge, no study has investigated the appetite of patients using validated screening tool after discharge from the ICU after 12 months.

Depression, a post-intensive care syndrome symptom, is a serious issue in patients after intensive care^7^. A systematic review reported that 29% of the patients experienced depression 12 months after ICU discharge^8^. To the best of our knowledge, no study has examined the relationship between depression and poor appetite in this population after intensive care.

Therefore, this study aimed to describe appetite loss using a validated screening tool to determine whether depression is associated with appetite loss, and to identify risk factors for appetite loss in older patients 12 months after ICU discharge.

## Methods

### Study design

This study was a secondary analysis of data obtained from a published ambidirectional study examining post-intensive care syndrome 12 months after discharge (SMAP-HoPe study) conducted in 12 ICUs in Japan^9^. This study focused on appetite in the older cohort.

Briefly, we conducted an ambidirectional study. Eligible patients were those who stayed in the ICU for at least 3 nights between October 2019 and July 2020 and were living at home for 12 months after discharge. The recruitment process continued every month until the specified sample size was reached. The exclusion criteria included central nervous disease, severe dementia, not living at home, and death 12 months after intensive care. After screening the medical chart, we made telephone calls to the candidate patients to clarify if they met the exclusion criteria. After confirming that the patients met the criteria, we sent a survey set including questionnaires regarding mental health and a Japanese-translated simplified nutritional appetite questionnaire (SNAQ) at 12 months after intensive care. The detailed recruitment process, study design, and characteristics of each institution have already been published^9^. A total of 754 participants living at home 12 months after intensive care were included in the previous study^9^. In this study we included only over 65 years old participants from SMAP-HoPe study.

### Participants

In this study, we included 501 participants who were over 65 years of age, among the 754 participants in the SMAP-HoPe study.

### Variables and Instruments

Variables including the Acute Physiology and Chronic Score II (APACHE II), diagnosis at ICU admission, ICU length of stay, and hospital length of stay were retrospectively recorded by a nurse researcher in each ICU. Digestive disease was defined as any digestive disease requiring intensive care, such as after esophageal resection, pancreaticoduodenectomy, and gastrointestinal perforation.

The SNAQ has been widely used to assess appetite and has been well validated^10^. A past study reported thatpatients with SNAQ scores of less than 14 developed significant weight loss after 6 months^10^. In subjects aged > 60 years, the sensitivity and specificity of the SNAQ for 10% weight loss were 83.3% and 77.6%, respectively^10^. The Japanese-translated SNAQ is reported to have sufficient validity and reliability for the ≥ 65 years community-dwelling population^11^. We defined poor appetite as < 14 on the SNAQ according to previous studies^10,12^.

The Hospital Anxiety and Depression Scale (HADS) is a commonly used, valid, and reliable questionnaire for assessing the degree of anxiety and depressive symptoms in outpatients^13^ and critically ill patients^14^. It has also been translated into Japanese, and its Japanese version has good reliability and validity^15^. The HADS consists of an anxiety subscale and a depression subscale, and each subscale has seven items rated on a scale of 0 to 3, with a total score of 0–21. Half of the items relate to anxiety symptoms, and the rest, to depressive symptoms. High correlations between HADS scores and psychiatric interview diagnoses of anxiety and depression have been reported (Spearman's correlation: r = 0.70 for anxiety severity, r = 0.74 for depression severity)^15^. In the Japanese version of the HADS, a score of 8 or higher was defined as substantial anxiety or depression^15^, and we followed this definition. In this study, the depression subscale of the HADS is referred to as HADS-D.

### Statistical analysis

Because this was a secondary analysis, the sample size was not calculated. Continuous variables and ordinary variables are presented as median and interquartile range [IQR], or as otherwise specified. Nominal variables are presented as percentages. The Kruskal–Wallis test or Fisher’s exact test was used to compare the two variables.

To clarify the relationship between poor appetite and severity of depression in the population, a multilevel generalized linear model with binomial family and logit links was used. The outcome variable was poor appetite (< 14 on the SNAQ). The covariates were chosen based on clinical plausibility and previous studies^16,17^^.^ The covariates included age, digestive disease, depression, and malignancy at admission. The results of multivariable analysis were presented as odds ratio (OR) and 95% confidential interval (95%CI).

The missing values of the HADS were imputed using the "half rule," which means that if half of the subscales were responded to, the mean value was imputed^18^. The missing items of the SNAQ were not imputed because they were considered missing completely at random. Thus, we excluded participants with missing items on the SNAQ. R software 4.0.2 (R Foundation for Statistical Computing, 2020) and Stata/IC16 (Stata Corp, TX) were used for analysis.

### Ethics information

The study was conducted in accordance with the guidelines of the Declaration of Helsinki and approved by the Institutional Review Board of Sapporo City University (No. 1927-1 on August 30th, 2019). Additionally, ethical approval was obtained from the ethics committees of all participating institutions. An explanatory document and consent form were sent to the study participants along with the study set. Informed consent was obtained from each participant prior to any study-related activities being performed.

## Results

### Characteristics of participants

**Figure 1:**
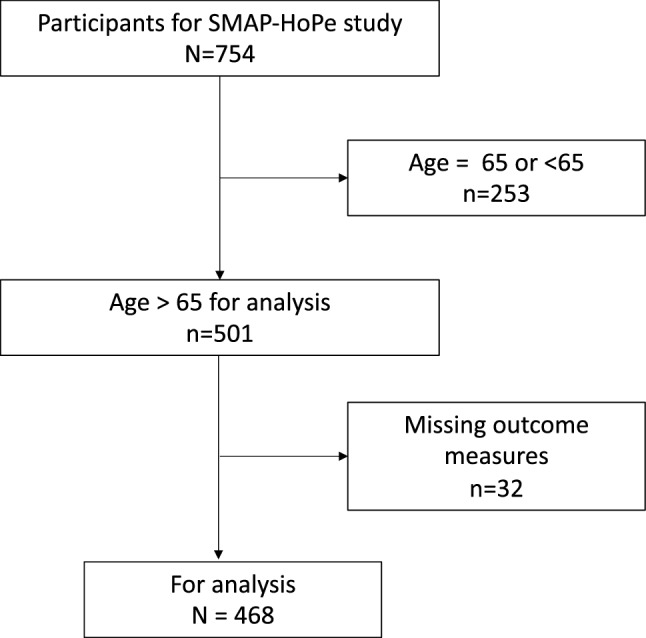
Flow diagram of this study.

Of the 501 older participants, 33 were excluded because of missing items on the SNAQ; 47 patients had partially missing HADS scores, which were imputed using the above method^18^. Thus, 468 participants were included in the analysis. The flow diagram is shown in Fig. 1. The characteristics of the study population are shown in Table 1. The median and IQR of age was 75.0 (71.0, 80.0) years, while the median (IQR) APACHE II was 15.00 [11.0, 20.0]. The number and proportion of participants with digestive disease was 58 of 468 (12.4%).

**Table 1:** The main characteristics data of the study.

Variables	N = 468
Age (years), median [IQR]	75.0 [71.0, 80.0]
Female, n (%)	142 (30.3)
Elective surgery, n (%)	236 (50.4)
Diagnosis at admission, n (%)	
CV surgery	218 (46.6)
CHF/AMI/Arry	72 (15.4)
Sepsis	49 (10.5)
Abdominal Surgery	41 (8.8)
Others	22 (4.7)
Acute Respiratory Failure	19 (4.1)
Aortic Dissection (Conservative Treatment)	16 (3.4)
ENT	15 (3.2)
Other Surgery	8 (1.7)
Trauma	8 (1.7)
Digestive disease, n (%)	58 (12.4)
APACHE II, median [IQR]	15.00 [11.0, 20.0]
MV use, n (%)	324 (69.2)
MV (days), median [IQR]	2.00 [0.0, 3.0]
Delirium, n (%)	122 (26.1)
Malignancy, n (%)	94 (20.1)
ICU LOS, median [IQR]	5.00 [4.0, 7.0]
Hospital LOS, median [IQR]	2.00 [0.0, 3.0]

IQR, interquartile range; CV, cardiovascular; CHF/AMI/Arrhy, congestive heart failure/acute myocardial infarction/arrhythmia; ENT, ear nose throat; APACHE II, Acute Physiology and Chronic Health Evaluation II; MV, mechanical ventilation; ICU, intensive care unit; LOS, length of stay.

### Appetite loss and depression symptom in the participants

The mean and standard deviation of SNAQ score was 14.2 ± 1.6, and the prevalence of poor appetite was 25.4% (95%CI, 21.529.4). The most frequent score was 14.

The median and IQR of HADS-D score was 5 [2–8], and 28.0% of the participants had substantial depression.

### Characteristics of participants with or without poor appetite

Table 2 shows a comparison of characteristics between groups, i.e., patients without poor appetite and those with poor appetite. The proportion of patients with malignancy at ICU admission was significantly higher among those with poor appetite than in those without poor appetite. Age, sex, severity of illness, proportion of subjects with digestive illness, and proportion of patients on mechanical ventilation were comparable between the two groups.

**Table 2:** Comparison of characteristics between groups.

Variables	Without poor appetite, n = 349	With poor appetite, n = 119	P value
Age (years), median [IQR]	75.0 [71.0, 80.0]	75.0 [70.0, 79.5]	0.855
Male (%)	244 (69.9)	82 (68.9)	0.908
Elective Surgery, n (%)	174 (49.9)	62 (52.1)	0.75
Diagnosis at admission, n (%)			0.83
CV surgery	165 (47.3)	53 (44.5)	
CHF/AMI/Arry	58 (16.6)	14 (11.8)	
Sepsis	35 (10.0)	14 (11.8)	
Abdominal surgery	27 (7.7)	14 (11.8)	
Others	17 (4.9)	5 (4.2)	
Acute respiratory failure	14 (4.0)	5 (4.2)	
ENT	12 (3.4)	4 (3.4)	
Aortic dissection (conservative treatment)	10 (2.9)	5 (4.2)	
Other surgery	6 (1.7)	2 (1.7)	
Trauma	5 (1.4)	3 (2.5)	
Digestive disease, n (%)	39 (11.2)	19 (16.0)	0.197
APACHE II, median [IQR]	15.0 [11.0, 20.0]	15.00 [11.0, 20.0]	0.815
MV use, n (%)	240 (68.8)	84 (70.6)	0.732
MV (days), median [IQR]	2.0 [0.0, 3.0]	2.00 [0.0, 3.0]	0.611
Delirium, n (%)	96 (27.5)	26 (21.8)	0.276
Malignancy, n (%)	62 (17.8)	32 (26.9)	0.035
ICU LOS, median [IQR]	5.0 [4.0, 7.0]	5.00 [4.0, 7.0]	0.981
Hospital LOS, median [IQR]	29.0 [20.0, 42.00]	28.0 [19.0, 39.5]	0.626

IQR, interquartile range; CV, cardiovascular; CHF/AMI/Arrhy, congestive heart failure/acute myocardial infarction/arrhythmia; Abd surgery, abdominal surgery; ENT, ear nose throat; APACHE II, Acute Physiology and Chronic Health Evaluation II; MV, mechanical ventilation; ICU, intensive care unit; LOS, length of stay.

### Multivariable analysis

**Figure 2:**
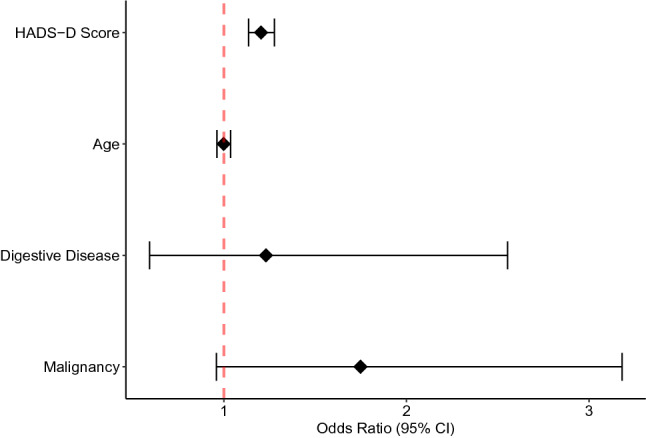
The results of multilevel generalized linear model with binomial family and logit link. Forest plot of odds ratio and 95% confidence intervals for adjusted variables. HADS-D shows the odds ratio for each one-point change in the score. Similarly, the odds ratio for a 1-year change in age is shown. HADS-D, Hospital Anxiety and Depression Scale–Depression.

**Figure 3:**
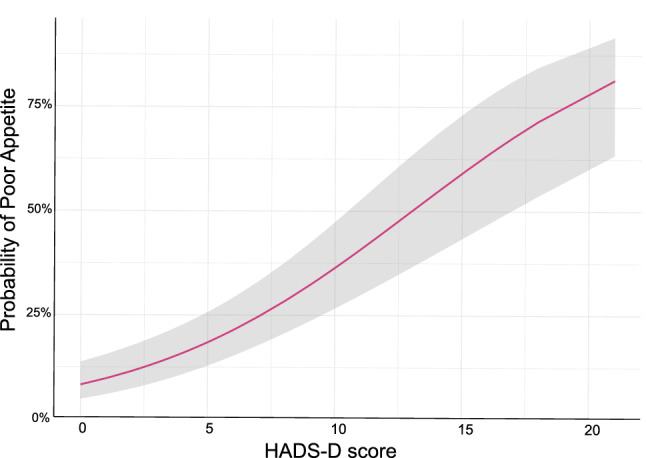
The relationship between severity of depression and risk of poor appetite after adjusting for age, HADS-D score, digestive disease, and malignancy as covariates, using a multilevel generalized linear model with binomial family and logit link. Grey areas show 95% confidence intervals. HADS-D is a subscale of the HADS that assesses depressive symptoms. A high HADS-D score indicates a high number of symptoms. Poor appetite was defined as a SNAQ score < 14. HADS, Hospital Anxiety and Depression Scale; HADS-D, Hospital Anxiety and Depression Scale—Depression subscale.

Figure 2 shows the forest plot of OR and 95%CI for each covariate after adjustment with multivariable analysis. High HADS-D score was an independent factor for a high probability of poor appetite (OR, 1.20; 95%CI, 1.14–1.28; p = 0.00). Age was not significantly associated with a poor appetite. Additionally, Fig. 3 shows the relationship between the severity of depressive symptoms and probability of appetite loss after adjusting for predefined covariates.

## Discussion

### Summary of main findings

We conducted a secondary analysis to clarify the prevalence of appetite loss and the relationship between appetite loss and severity of depression in the older patients who were living at home 12 months after intensive care. One in four patients experienced significant appetite loss, and the severity of depression was an independent factor contributing to appetite loss.

### Comparison of current and previous results

In this study, appetite loss was more common in patients who were living at home 1 year after discharge from the ICU than in the general community-dwelling elderly and this was comparable to that in rehabilitation wards. In one study of 9496 elderly Japanese, the prevalence of appetite loss (SNAQ score < 14) was 9.8%^3^. In another report, 24.7% of persons aged 65 years or older in a rehabilitation ward had a SNAQ score < 14^12^, which was comparable to that reported in our study.

Appetite should be focused on while assessing the health of ICU survivors. Studies of long-term appetite loss after intensive care at 1 year are few, making comparisons difficult. Past studies showing loss of appetite at 6 months after ICU discharge compared to before ICU admission suggests that loss of appetite may persist beyond 6 months^11^. Additionally, other cohort study suggested that lower appetite after ICU discharge compared with age-matching healthy population^6^. These results of the past study were consistent with our study; however, the past studies did not use a validated screening tool^5,19^. We consider that our results provide increased confidence that loss of appetite will persist long after intensive care since we used a validated screening tool.

The underlying mechanisms of decreased appetite after intensive care remain unclear. In a previous study, appetite loss was associated with C-reactive protein (CRP) levels immediately and 1 week after intensive care, but after 3 months, appetite loss was no longer associated with CRP levels^20^. Therefore, it is unlikely that CRP levels affected appetite status 1 year after intensive care.

Similarly, the underlying mechanism leading to appetite loss for long term ICU survivors is unclear; intervention to improve appetite loss is also unknown. A randomized controlled study suggested that enhanced rehabilitation including dietetic assessment after ICU discharge failed to improve appetite loss at 3 months after ICU discharge^21^. Further investigation to clarify mechanism of appetite loss after intensive care was warranted.

### Relationship between poor appetite and depression

Severity of depression may be associated with a high probability of appetite loss in our population. This finding is consistent with that of a previous cross-sectional study, where appetite loss was associated with a high probability of depression in community-dwelling older people and people living in assisted facilities^22,23^. Depression is a common psychological problem and an important co-symptom of decreased appetite and decreased motivation to eat in older adults; however, the underlying mechanism is unclear^24^. A high prevalence of depression has been reported in post-intensive care patients^8^; therefore, we should focus on appetite loss as well as depression. This may contribute to the improvement in the quality of life of patients discharged from the ICU.

### Strengths and Limitations

To the best of our knowledge, this is the first study to use a validated tool to investigate appetite status 1 year after ICU discharge; however, some limitations exist. The characteristics of the ICU patients (i.e., a high proportion were scheduled for surgery) reflected the findings. Thus, different populations of patients admitted to the ICU may have led to different results. However, appetite loss appeared to be independent of the severity of illness or reason for ICU admission; thus, we do not consider this aspect to have a significant impact on external validity. In this study, we measured appetite loss using the SNAQ; however, the prevalence rates of actual weight loss, sarcopenia, and frailty were not determined. Furthermore, investigation was ‘not conducted to determine whether appetite loss or sarcopenia was present prior to ICU admission.

Further studies are needed to clarify the relationships between appetite loss and frequency of sarcopenia after intensive care in a large prospective cohort. Additionally, the present study did not measure patients’ weight. The relevance of the patients' weight to appetite loss would require further clarification. Moreover, we clarified that appetite loss was associated with a high severity of depression; however, the causal relationship was unclear because of our study design. A study design that measures one of the exposed factors prior to outcome is needed.

### Clinical implications

In post-ICU older adults, in addition to screening for mental health, screening for appetite loss should also be performed. In addition to physical examinations, adequate nutritional counseling may help in the early detection and improvement of nutritional status and may help prevent sarcopenia.

## Conclusion

Poor appetite is a common finding 12 months after ICU admission. This may lead to sarcopenia and a high mortality rate. Additionally, poor appetite is associated with a high severity of depression. It is necessary to monitor and treat these symptoms when observed after intensive care.

## Data Availability

The data that support the findings of this study are available upon request from the corresponding author (T.U.). The data are not publicly available because they contain information that can compromise the privacy of the research participants.
